# Supplementary material for: FOXD1 Repression Potentiates Radiation Effectiveness by Downregulating G3BP2 Expression and Promoting the Activation of TXNIP-Related Pathways in Oral Cancer
Source: Cancers (Basel). 2020 Sep 21;12(9):2690. doi: 10.3390/cancers12092690 (PMC7563336; doi:10.3390/cancers12092690)
Supplement: Supplementary file 1 [file cancers-12-02690-s001.pdf]

cancers

MDPI

Article

FOXD1 Repression Potentiates Radiation Effectiveness by Downregulating *G3BP2* Expression and Promoting the Activation of *TXNIP*-Related Pathways in Oral Cancer

Che-Hsuan Lin [1,2,†], Hsun-Hua Lee [3,4,5,6], Wei-Min Chang [7], Fei-Peng Lee [1,8], Lung-Che Chen [1,2,†], Long-Sheng Lu [9,10] and Yuan-Feng Lin [3,11,*]

1 Department of Otolaryngology, School of Medicine, College of Medicine, Taipei Medical University, Taipei 11031, Taiwan; cloudfrank@gmail.com (C.-H.L.); fplee@tmu.edu.tw (F.-P.L.)
2 Department of Otolaryngology, Taipei Medical University Hospital, Taipei Medical University, Taipei 11031, Taiwan; b101093017@tmu.edu.tw (L.-C.C.)
3 Graduate Institute of Clinical Medicine, College of Medicine, Taipei Medical University, Taipei 11031,Taiwan; kaorulei@yahoo.com.tw (H.-H.L.); d001089012@tmu.edu.tw (Y.-F.L.)
4 Department of Neurology, Shuang Ho Hospital, Taipei Medical University, New Taipei City 235, Taiwan
5 Department of Neurology, School of Medicine, College of Medicine, Taipei Medical University, Taipei 11031, Taiwan
6 Department of Neurology, Vertigo and Balance Impairment Center, Shuang Ho Hospital, Taipei Medical University, New Taipei City 235, Taiwan
7 School of Oral Hygiene, College of Oral Medicine, Taipei Medical University, Taipei 11031, Taiwan; weiminchang@tmu.edu.tw (W.-M.C.)
8 Department of Otolaryngology, Shuang-Ho Hospital, Taipei Medical University, New Taipei City 235, Taiwan
9 Department of Radiation Oncology, Taipei Medical University Hospital, Taipei Medical University, Taipei 11031, Taiwan; lslu@tmu.edu.tw (L.-S.L.)
10 Graduate Institute of Biomedical Materials and Tissue Engineering, College of Biomedical Engineering, Taipei Medical University, Taipei 11031, Taiwan
11 Cell Physiology and Molecular Image Research Center, Wan Fang Hospital, Taipei Medical University, Taipei 11696, Taiwan
* Correspondence: d001089012@tmu.edu.tw; Tel.: +886-2-2736-1661 (ext. 3106)
† The authors equally contributed to this study.

Received: 15 September 2020; Accepted: 16 September 2020; Published: date

Supplementary materials

Table 1. The list of upregulated (log2FC ≥ 1.0) and downregulated (log2FC ≤ −1.0) genes after FOXD1 knockdown in A375 and MeWo cells.

**Upregulated genes ($log_2FC \geq 1.0$)**					**Downregulated genes ($log_2FC \leq -1.0$)**				
**Gene Symbol**	**A375**		MeWo		Gene Symbol	A375		MeWo	
	**log_2FC**	**adj. p value**	log_2FC	adj. p value		log_2FC	adj. p value	log_2FC	adj. p value
FAM46A	1.86	3.47E-03	1.51	3.28E-03	FOXD1	−2.37	5.96E-06	−2.45	1.04E-05
STK40	1.64	5.96E-06	1.20	1.37E-03	G3BP2*	−2.02	1.80E-07	−1.85	1.25E-03
ID3	1.60	1.55E-03	1.22	9.53E-03	DCP2	−1.67	7.27E-07	−1.54	1.25E-03
CD163L1	1.50	3.47E-03	1.15	3.81E-03	CCL2	−1.63	1.84E-03	−1.17	2.07E-03
TXNIP	1.42	1.73E-02	1.57	1.49E-03	ANGPTL4	−1.59	1.29E-03	−1.27	1.14E-02
CD163L1	1.33	4.99E-03	1.11	1.56E-03	LEPROTL1	−1.54	1.28E-05	−1.55	2.07E-03
MAGEB2	1.31	6.74E-05	1.51	1.37E-03	G3BP2*	−1.52	2.14E-06	−1.30	1.49E-03
PANX2	1.29	1.62E-04	1.01	3.75E-03	TGM2	−1.47	6.92E-04	−1.50	7.49E-03
AKR1C2	1.15	7.66E-03	1.25	2.19E-03	CLDN12	−1.46	1.53E-05	−1.33	1.49E-03
COX7B2	1.13	4.85E-03	1.33	3.58E-03	PRKAA1	−1.45	6.56E-06	−1.35	2.19E-03
SLC25A44	1.12	1.96E-04	1.11	5.42E-03	FAM216A	−1.33	3.03E-05	−1.42	1.25E-03

BCAT2	1.08	4.25E-05	1.21	1.49E-03	G3BP2*	−1.30	1.27E-04	−1.49	7.10E-04
SIDT2	1.08	3.49E-03	1.26	1.49E-03	CTDSPL	−1.28	4.99E-05	−1.15	1.92E-03
HIST1H2BD	1.06	1.69E-01	1.02	8.54E-03	TMEM245	−1.25	4.95E-05	−1.22	1.92E-03
AKR1C4	1.01	6.83E-02	1.17	1.92E-03	RRM2	−1.23	1.27E-04	−1.09	4.54E-03
SEL1L3	1.01	1.59E-03	1.44	1.49E-03	ESM1	−1.22	1.57E-02	−1.63	4.22E-03
					LRRC20	−1.22	1.72E-03	−1.16	1.25E-02
					NTM	−1.22	2.16E-05	−1.07	2.19E-03
					SLC16A3	−1.17	2.47E-04	−1.36	1.52E-02
					BGN	−1.17	3.91E-03	−1.25	3.40E-03
					AMMECR1	−1.16	1.58E-04	−1.03	5.42E-03
					GPR180	−1.14	1.28E-05	−1.18	2.82E-03
					ANKRD1	−1.14	7.62E-03	−1.07	5.42E-03
					MBNL1	−1.12	9.62E-04	−1.03	6.55E-03
					VSTM1	−1.10	5.53E-03	−1.08	2.31E-02
					RBPMS2	−1.08	3.03E-05	−1.00	1.14E-02
					STYX	−1.06	4.71E-04	−1.11	2.46E-03
					EVA1A	−1.05	6.99E-05	−1.01	2.07E-03
					SLBP	−1.03	3.03E-05	−1.11	1.49E-03
					RFTN1	−1.02	1.29E-03	−1.10	3.45E-03

*The values of log2FC dertermined by three independent G3BP2 probes ILMN_2381753, ILMN_2381758 and ILMN_1720422 in Illumina Human HT-12 V4.0 expression beadchip. The adj. p value denotes the Benjamini & Hochberg false discovery rate (FDR) adjusted p-value in GEO2R analysis.

Table 2. The list of candidate pathways from GSEA experiment.

**HALLMARK Gene sets**	**A375**				**MeWo**			
**NAME**	**ES**	**NES**	**NOM p-val**	**FDR q-val**	**ES**	NES	NOM p-val	FDR q-val
INTERFERON_ALPHA_RESPONSE	0.61	2.40	0.00	0.00	0.56	2.27	0.00	0.00
OXIDATIVE_PHOSPHORYLATION	0.42	1.80	0.00	0.00	0.39	1.71	0.00	0.00
INTERFERON_GAMMA_RESPONSE	0.38	1.68	0.00	0.01	0.33	1.47	0.00	0.03
ANGIOGENESIS	0.50	1.67	0.01	0.01	0.56	1.88	0.00	0.00
P53_PATHWAY	0.36	1.56	0.00	0.01	0.45	1.99	0.00	0.00
XENOBIOTIC_METABOLISM	0.34	1.48	0.00	0.02	0.34	1.52	0.00	0.02
PROTEIN_SECRETION	0.37	1.45	0.01	0.03	0.43	1.73	0.00	0.01
SPERMATOGENESIS	−0.39	−1.42	0.02	0.05	−0.47	−1.71	0.00	0.00
CHOLESTEROL_HOMEOSTASIS	−0.42	−1.43	0.03	0.05	−0.47	−1.60	0.00	0.01
ESTROGEN_RESPONSE_LATE	−0.39	−1.51	0.01	0.02	−0.47	−1.78	0.00	0.00
DNA_REPAIR	−0.41	−1.52	0.00	0.02	−0.47	−1.72	0.00	0.00
GLYCOLYSIS	−0.44	−1.68	0.00	0.00	−0.38	−1.44	0.01	0.04
ESTROGEN_RESPONSE_EARLY	−0.45	−1.73	0.00	0.00	−0.43	−1.66	0.00	0.00
HYPOXIA	−0.47	−1.77	0.00	0.00	−0.39	−1.48	0.00	0.02
UNFOLDED_PROTEIN_RESPONSE	−0.54	−1.91	0.00	0.00	−0.50	−1.76	0.00	0.00
MITOTIC_SPINDLE	−0.50	−1.94	0.00	0.00	−0.52	−2.01	0.00	0.00
MTORC1_SIGNALING	−0.53	−2.04	0.00	0.00	−0.51	−1.93	0.00	0.00
MYC_TARGETS_V2	−0.65	−2.10	0.00	0.00	−0.67	−2.14	0.00	0.00
MYC_TARGETS_V1	−0.59	−2.24	0.00	0.00	−0.62	−2.35	0.00	0.00
G2M_CHECKPOINT	−0.69	−2.66	0.00	0.00	−0.75	−2.83	0.00	0.00
E2F_TARGETS	−0.72	−2.74	0.00	0.00	−0.80	−3.02	0.00	0.00

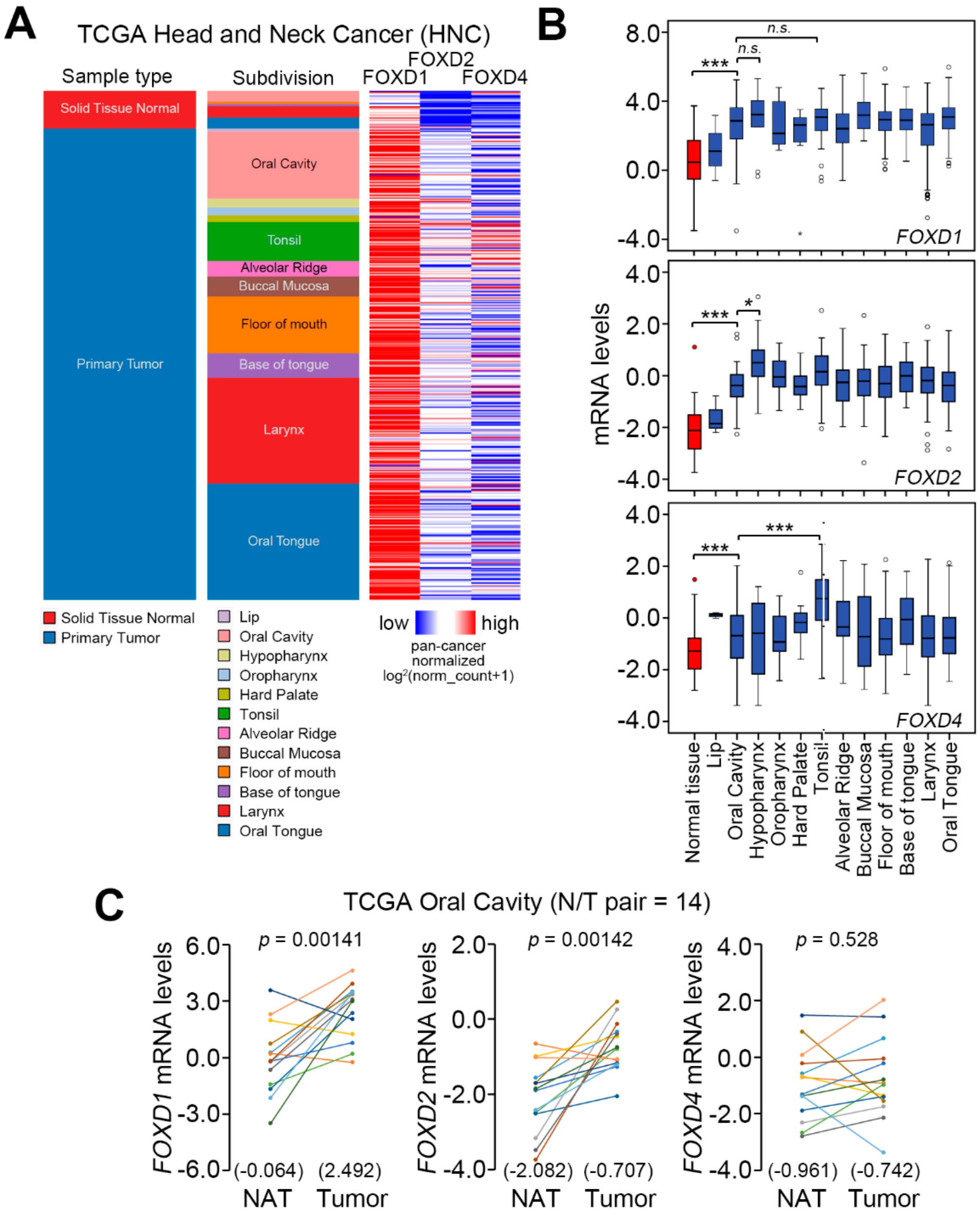

Figure 1. The transcriptional profile of FOXD1, FOXD2 and FOXD4 in the anatomic subdivision of TCGA head and neck cancer. (**A** and **B**) The heatmap (**A**) and boxplot (**B**) for the mRNA levels of FOXD1, FOXD2 and FOXD4 in the normal tissues and primary tumors derived from the different anatomic subdivision of TCGA head and neck cancer. The statistical differences were analyzed by student t-test. (**C**) The mRNA levels of FOXD1, FOXD2 and FOXD4 in the normal adjacent tissues (NAT) and primary tumors derived from oral cavity using TCGA head and neck cancer database. The statistical significances were evaluated by paired t-test.

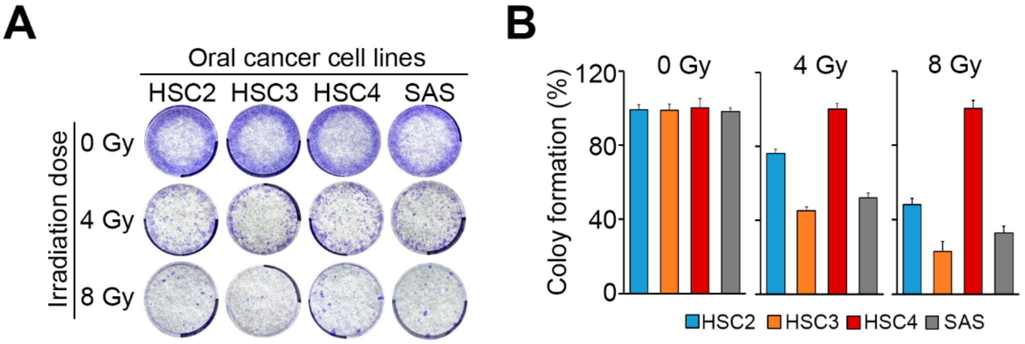

Figure 2. The colony-forming ability of HSC2, HSC3, HSC4 and SAS oral cancer cells after irradiation exposure at the designated doses. (**A** and **B**) Crystal violet staining for the cell colonies of HSC2, HSC3, HSC4 and SAS oral cancer cells at 2 weeks post-exposure to the designated dose of irradiation (A) and the histograms for the results obtained from three independent experiments of colony-forming assay (**B**).

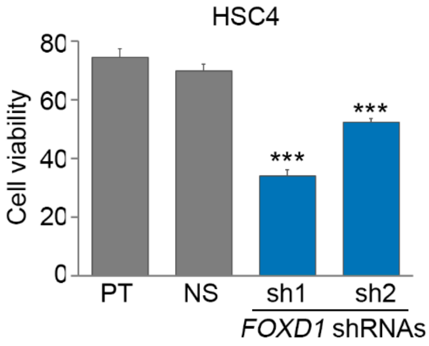

Figure 3. FOXD1 knockdown sensitizes HSC4 cells to irradiation treatment. Cell viability of parental (PT) HSC4 cells and HSC4 cells transcfected with non-silencing (NS) control shRNA or 2 independent FOXD1 shRNAs at 24 hours post-exposure to 8 Gy irradiation. The data obtained from three independent experiment presented as mean ± SEM. Non-parametric Friedman test was used to estimate the statistical significances. The symbol "***" denotes statistical p value < 0.001.

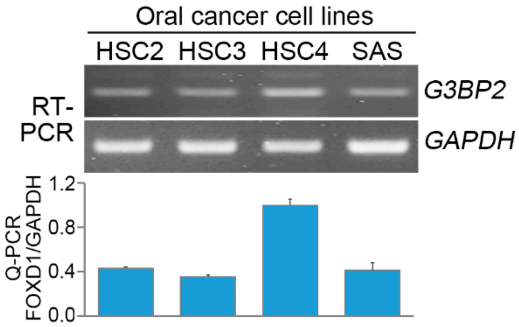

Figure 4. The measurement of G3BP2 mRNA levels in oral cancer cell lines. The mRNA levels of G3BP2 and GAPDH detected by RT-PCR and Q-PCR experiments in a panel of oral cancer cell lines HSC-2, HSC-3, HSC-4 and SAS. GAPDH was used as an internal control of RT-PCR and Q-PCR experiment.

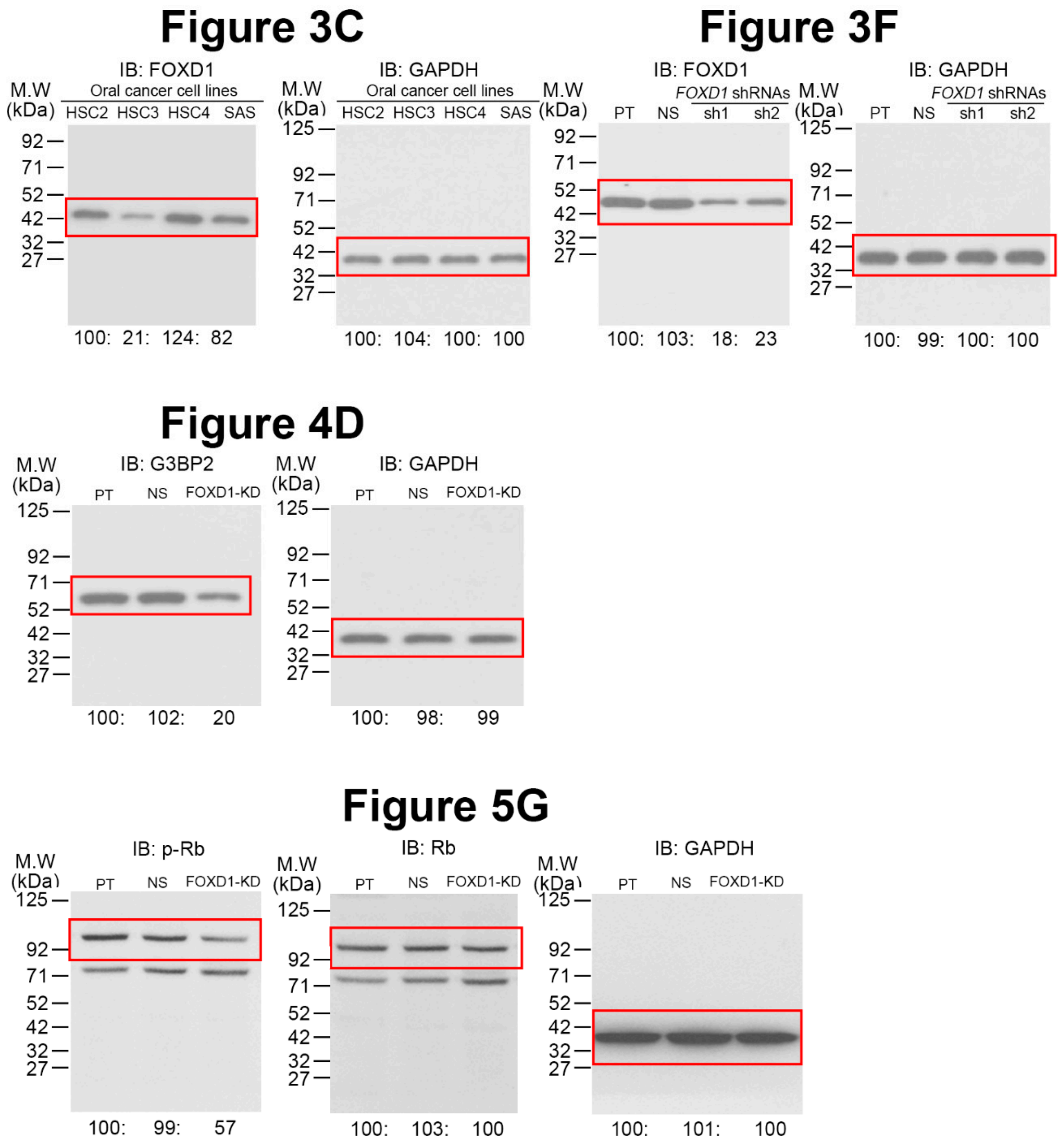

Figure 5. Uncut blots for Figure 3C, 3F, 4D and 5G.
